# CD147 functions as the signaling receptor for extracellular divalent copper in hepatocellular carcinoma cells

**DOI:** 10.18632/oncotarget.17712

**Published:** 2017-05-09

**Authors:** Pengfei Ding, Xin Zhang, Shujuan Jin, Bo Duan, Pengxiang Chu, Yufei Zhang, Zhi-Nan Chen, Bin Xia, Fei Song

**Affiliations:** ^1^ Beijing Nuclear Magnetic Resonance Center, School of Life Sciences, College of Chemistry and Molecular Engineering, Peking University, Beijing 100871, China; ^2^ Department of Cell Biology, State Key Laboratory of Cancer Biology, Cell Engineering Research Center, The Fourth Military Medical University, Xi’an 710032, China

**Keywords:** CD147, extracellular divalent copper, matrix metalloproteinase, invasion, hepatocellular carcinoma

## Abstract

Elevated copper levels in tumor microenvironment are directly correlated to cancer progression in a variety of malignancies. Copper is required in angiogenesis, and promotes the proliferation and metastasis of cancer cells. However, the molecular mechanism of copper in promoting cancer progression remains elusive. Here we report that CD147 serves as a signaling receptor for extracellular Cu^2+^ in hepatocellular carcinoma (HCC) cells. Cu^2+^ binds to the extracellular membrane-proximal domain of CD147 and mediates its self-association. Cu^2+^-mediated self-association of CD147 activates PI3K/Akt signaling pathway leading to the up-regulation of matrix metalloproteinase MMP-2 and MMP-14 in HCC cells. Cu^2+^-induced CD147 self-association also enhances the ability of HCC cells to stimulate MMP-2 expression from neighboring fibroblasts, as well as increases the invasiveness of HCC cells which is abolished by the copper chelator tetrathiomolybdate. We have mapped the interfaces and identified the key residues of CD147 involved in the Cu^2+^ induced self-association. The Cu^2+^ binding deficient CD147 mutant abolishes the stimulating effects of Cu^2+^ on HCC cells. Our study reveals a novel extracellular signaling role of copper in promoting cancer cell metastasis, which implies that targeting the Cu^2+^-induced self-association of CD147 is a new strategy for cancer treatment.

## INTRODUCTION

Copper, an essential transition metal for life, serves as an important structural and catalytic cofactor involved in a diverse series of biochemical processes [1]. Elevation of copper in serum and cancer tissues are found in patients with a wide variety of malignancies, such as lymphoma, reticulum cell sarcoma, cervical, stomach, ovarian, lung, prostate, breast and liver cancer, and is directly correlated to cancer progression [2]. Copper has long been known to promote angiogenesis [3], and was shown to directly stimulate proliferation and invasion of cancer cells [4, 5]. Over a dozen clinical trials have been carried out for copper-lowering anti-cancer therapies [6–10], and it was recently reported that copper depletion by a potent copper chelator tetrathiomolybdate (TM) shows a striking effect in the treatment of triple negative breast cancer in a phase II clinical trial [11, 12].

Up to date, the molecular mechanism of copper in promoting cancer progression is still poorly understood. A large number of pro-angiogenic factors have been shown to be copper dependent [13]: copper may directly bind to angiogenic growth factors, such as angiogenin, and increase their affinity for endothelial cells [14]; copper may control the secretion of angiogenic cytokines, as demonstrated with bFGF and IL-1α [15]; copper may induce the expression of angiogenic growth factor such as VEGF [16, 17]. However, the direct causative connection and clear molecular basis for the high sensitivity of angiogenesis to copper has not been reached. For other stimulating effects of copper on cancer cells, studies have mainly focused on the roles of intracellular copper as cofactors of enzymes [18]. Intracellular copper is the catalytic cofactor of Memo which plays a critical role in cell motility [19]. It was recently reported that intracellular copper is required for oncogenic BRAF signaling and tumorigenesis [20]. On the other hand, it was found that extracellular divalent copper (Cu^2+^) is essential for the activities of both lysyl oxidase and lysyl oxidase-like proteins, which are directly related to cancer cell metastasis [21]. A recent study elegantly showed that the elevation of copper in blood of hepatocellular carcinoma (HCC) patients is not of exogenous origin, and is suggested to be released from intracellular copper storages [22]. Furthermore, it was found that 80–90% of intracellular copper are translocated into the extracellular space of the leading edge of endothelial cell projections during angiogenesis [23]. These redistributed copper ions were suggested to bind to and activate yet unknown cell surface receptors or extracellular targets [3].

CD147 (also known as Basigin) is a type I transmembrane glycoprotein with two immunoglobulin (Ig)-like domains [24–28]. It is widely expressed in human tissues and has been implicated in many physiological and pathological processes [29, 30]. Particularly, CD147 is highly-enriched on the surface of a wide variety of malignant tumor cells, including all the cancers with elevated copper levels described above [30]. CD147 plays very important roles in angiogenesis, proliferation and metastasis of many cancers and is an effective diagnostic marker and therapeutic target [30–32], especially in HCC treatment using CD147-directed monoclonal antibodies [33]. The biological functions of CD147 involved in cancer progression are largely attributed to its ability to stimulate the secretion of extracellular matrix metalloproteinases (MMPs, a family of zinc-dependent endopeptidases) in cancer cell themselves and from neighboring fibroblasts [34]. MMP-mediated extracellular matrix degradation greatly contributes to cancer cell invasion and metastasis [35]. Although the MMP-inducing activity of CD147 is suggested to be dependent on its self-association [34], the reported homophilic interaction interfaces are controversial and little is known about the regulatory mechanisms involved in this process [36–38]. Therefore, the molecular mechanism of CD147 in stimulating the secretion of MMPs has not been fully elucidated.

Here we establish a direct link for the functions of extracellular Cu^2+^ and CD147 by demonstrating that Cu^2+^ plays a signaling role on HCC cells through mediating the self-association of its cell surface receptor CD147, which in turn promotes MMPs secretion and enhances the invasiveness of HCC cells.

## RESULTS

### Extracellular Cu^2+^ up-regulates MMP-2 and MMP-14 expression in HCC cells through activating PI3K/Akt signaling pathway

**Figure 1:**
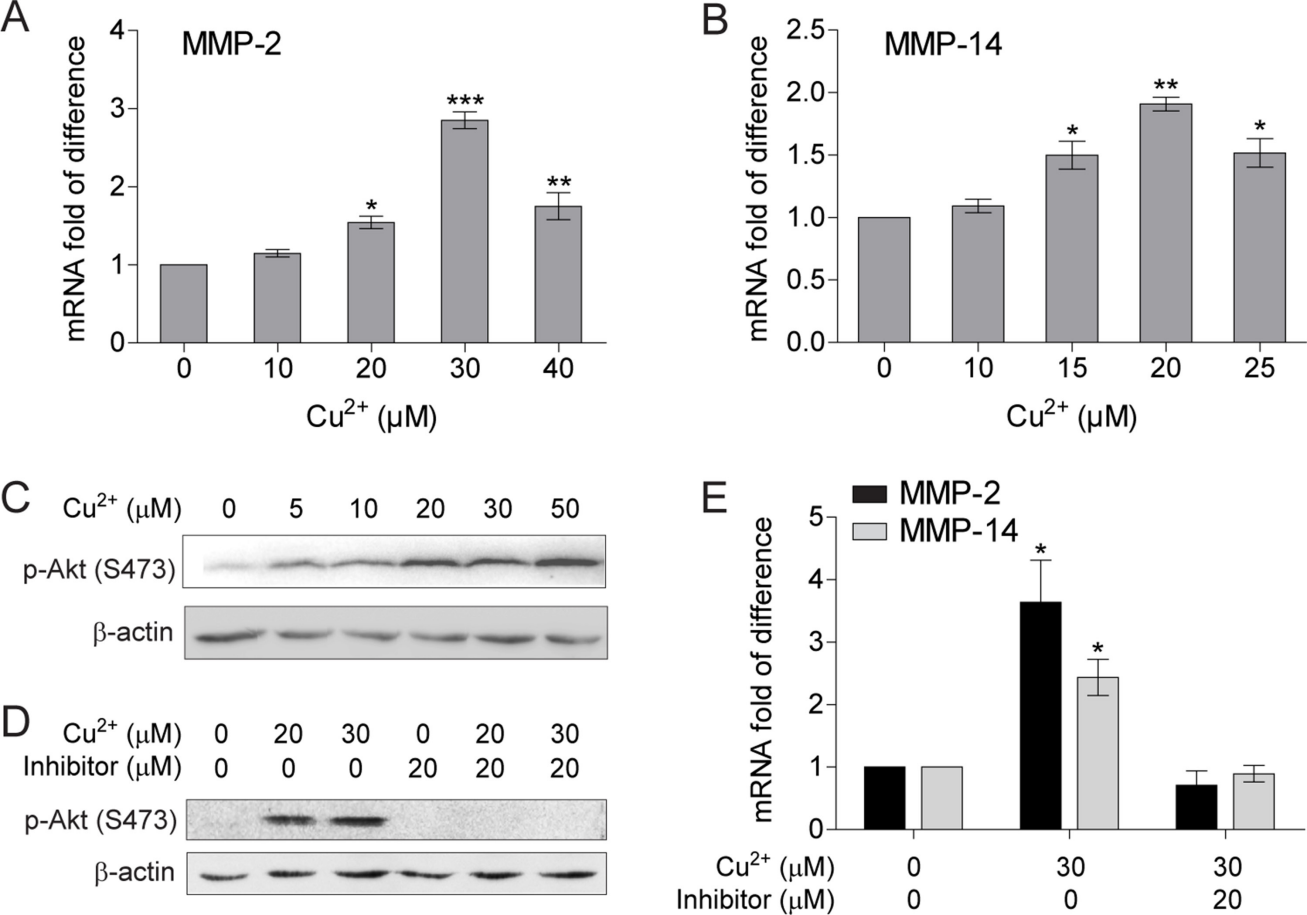
Cu^2+^ stimulates MMP-2 and MMP-14 expression in HCC cells through activating PI3K/Akt signaling pathway (**A**, **B**) qRT-PCR analysis of MMP-2 (A) and MMP-14 (B) in SMMC-7721 cells treated with different concentrations of Cu^2+^ (*n* = 3, mean ± SEM, one way ANOVA). (**C**) Immublot analysis of the p-Akt levels in SMMC-7721 cells treated with different concentrations of Cu^2+^. (**D**) PI3K inhibitor (LY294002) reduces the Cu^2+^-augmented p-Akt levels in SMMC-7721 cells. (**E**) PI3K inhibitor abolishes Cu^2+^-induced mRNA elevation of MMP-2 and MMP-14 (*n* = 3, mean ± SEM, one way ANOVA). *P < 0.05, **P < 0.01 and ***P < 0.001. Gel images in panel C and D are representative of at least two technical replicates.

The copper chelator tetrathiomolybdate (TM) was shown to inhibit tumor cell metastasis through down-regulating MMPs levels [39]. We used quantitative PCR with reverse transcription (qRT-PCR) to verify whether copper is able to directly stimulate MMPs expression in cancer cells. The mRNA levels of both MMP-2 and MMP-14 in human hepatocellular carcinoma (HCC) cell SMMC-7721 were up-regulated by Cu^2+^ in a concentration dependent manner, whereas the mRNA levels for other tested MMPs (MMP-1, MMP-3 and MMP-9) were not changed when treated with up to 40 μM Cu^2+^ (Figure 1A and 1B, Supplementary Figure 1). As shown in Figure 1, MMP-2 mRNA level reaches a maximum at 30 μM Cu^2+^, while the maximum for MMP-14 is at 20 μM Cu^2+^. The membrane-anchored MMP-14 also functions as the activator of secreted MMP-2 from its inactive zymogen; and both MMP-2 and MMP-14 promotes cancer cell invasion and metastasis [35, 40, 41], implicating that Cu^2+^ may modulate the metastatic properties of HCC cells by directly up-regulating MMP-2 and MMP-14 expression.

Copper has been shown to strongly activate the phosphoinositide 3 kinase (PI3K)/Akt signaling both in normal and cancer cells [42, 43]. Activation of PI3K/Akt signaling was also reported to be involved in MMP up-regulation in HCC cells [44]. We thus examined whether the MMP-inducing activity of copper is dependent on PI3K/Akt signaling pathway. The amount of phosphorylated Akt (p-Akt) in SMMC-7721 cells was significantly increased by Cu^2+^ at a concentration of 5 μM, which was further augmented by Cu^2+^ with concentrations higher than 20 μM (Figure 1C). Interestingly, Cu^2+^-stimulated expressions of MMP-2 and MMP-14 were abolished when the phosphorylation of Akt was inhibited by the specific PI3K inhibitor LY294002 (Figure 1D and 1E). These results suggest that activation of PI3K/Akt signaling is essential for Cu^2+^ to up-regulate MMP-2 and MMP-14 expression in HCC cells.

**Figure 2:**
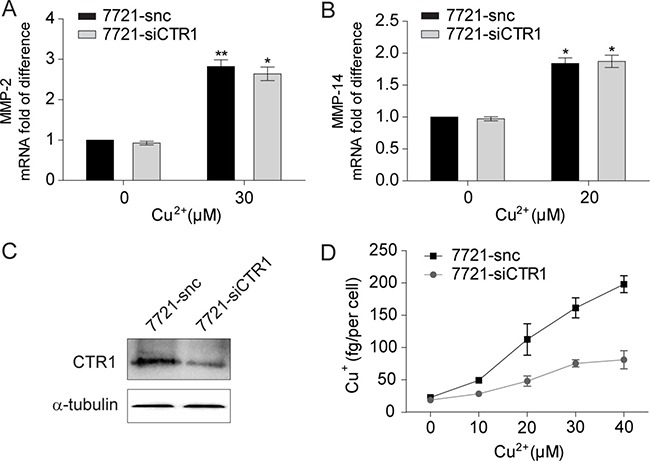
Intracellular uptake is not required for copper to up-regulate MMP-2 and MMP-14 expression (**A**, **B**) qRT-PCR analysis of MMP-2 (A) and MMP-14 (B) in SMMC-7721 cells with or without Cu^2+^ treatment (*n* = 3, mean ± SEM, *t*-test). Cells were transfected with ctr1-specific siRNA (7721-siCTR1) or control siRNA (7721-snc). (**C**) The copper intracellular uptake was significantly impaired in 7721-siCTR1 cells compared with 7721-snc cells as assessed by atomic absorption spectroscopy (*n* = 2, mean ± SEM). (**D**) Immunoblot analysis of CTR1 in 7721-siCTR1 and 7721-snc cells. *P < 0.05 and **P < 0.01. Gel images in panel D are representative of at least two technical replicates.

We next investigated whether decreasing the influx of copper affects its stimulation of MMPs expression in SMMC-7721 cells. Knockdown of CTR1, the key transporter for cellular copper uptake, had no effect on the copper-induced elevation of MMP-2 and MMP-14 mRNA levels (Figure 2A–2C), even though the intracellular monovalent copper (Cu^+^) concentration was significantly decreased (Figure 2D). Therefore, it is the extracellular divalent Cu^2+^, rather than the intracellular monovalent Cu^+^, that up-regulates MMP-2 and MMP-14 expression in HCC cells.

### CD147 is indispensible for Cu^2+^-stimulated up-regulation of MMPs

**Figure 3:**
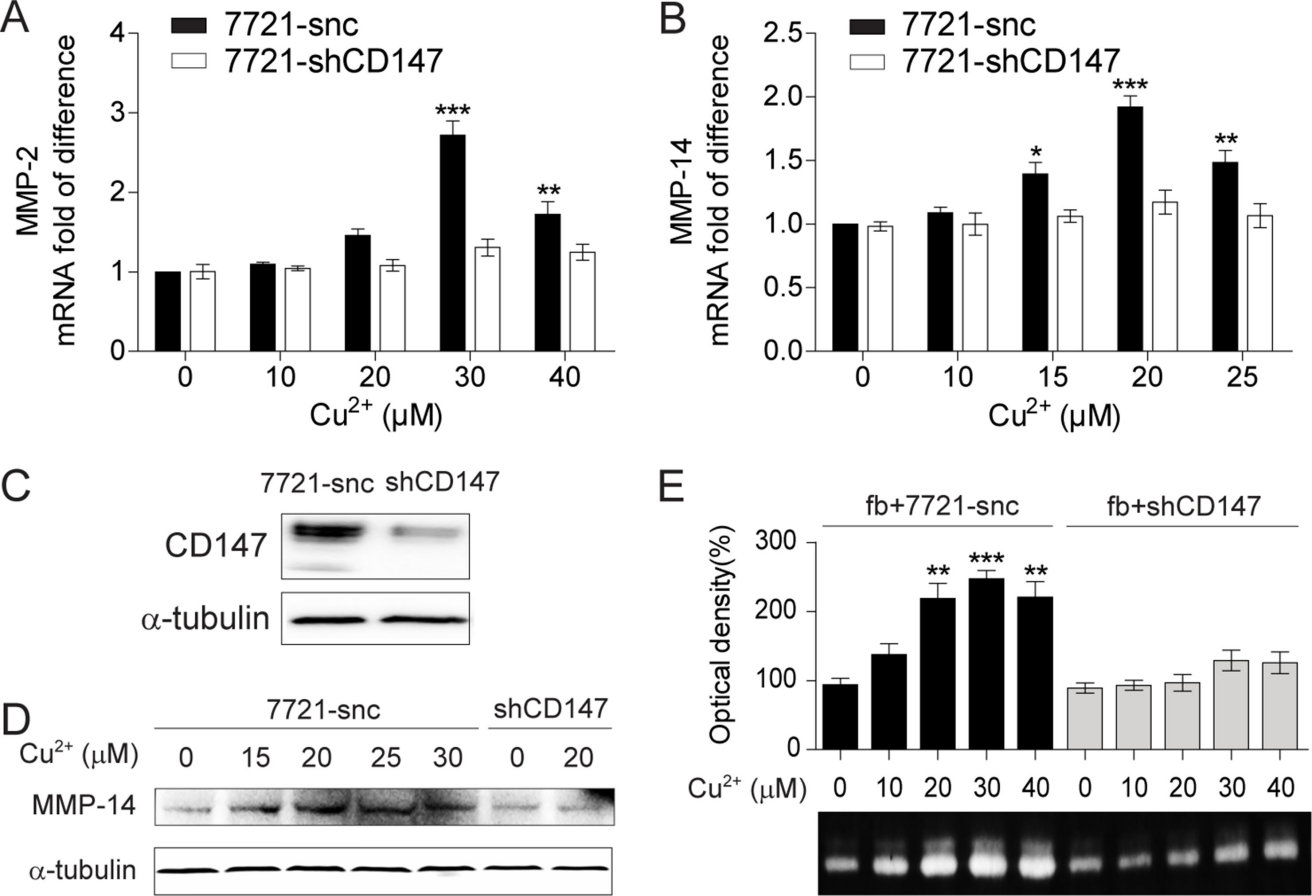
The MMP-inducing activity of Cu^2+^ is CD147 dependent (**A**, **B**) qRT-PCR analysis of MMP-2 (A) and MMP-14 (B) in SMMC-7721 cells treated with different concentrations of Cu^2+^ (*n* = 3, mean ± SEM, one way ANOVA). Cells were transfected with *cd147*-specific shRNA (7721-shCD147) or control shRNA (7721-snc). (**C**) The expression of CD147 is knocked down in 7721-shCD147 but not 7721-snc cells. (**D**) Immunoblot analysis of MMP-14 in 7721-snc or 7721-shCD147 cells treated with different concentrations of Cu^2+^. (**E**) The levels of MMP-2 secreted from fibroblasts (fb) co-cultured with 7721-snc or 7721-shCD147 cells as determined by gelatin zymography (*n* = 3, mean ± SEM, one way ANOVA). *P < 0.05, **P < 0.01 and ***P < 0.001. Gel images in panel C and D are representative of at least two technical replicates.

As CD147 is well-characterized as an inducer of MMPs [31, 32], we thus investigated whether CD147 is involved in MMPs expression stimulated by extracellular Cu^2+^. It was found that the Cu^2+^-induced up-regulation of MMP-2 and MMP-14 mRNA levels were markedly decreased when the expression of CD147 was suppressed by short hairpin RNA (shCD147) (Figure 3A–3C). Immunoblot showed that knockdown of CD147 also impaired the elevated MMP-14 protein level by Cu^2+^ treatment (Figure 3D). Thus, the up-regulation of MMP-2 and MMP-14 expression in HCC cells by extracellular Cu^2+^ is CD147 dependent.

Over-expressed CD147 on cancer cell surface strongly activate MMPs production from adjacent fibroblasts [34]. We asked whether Cu^2+^ also plays a stimulatory role in this process. Gelatin zymography showed that Cu^2+^ treatment stimulated MMP-2 secretion from fibroblasts co-cultured with SMMC-7721 cells, which was notably impaired by the knockdown of CD147 (Figure 3E). MMP-2 expression was not affected by Cu^2+^ treatment when fibroblasts were cultured alone (Supplementary Figure 2). These results suggest that Cu^2+^ increases the capability of HCC cells to induce MMP-2 expression from neighboring fibroblasts in a CD147 dependent manner.

However, Cu^2+^ treatment did not alter the expression level of CD147 in SMMC-7721 cells (Supplementary Figure 3), suggesting that the up-regulation of MMPs expression in HCC cells or neighboring fibroblasts is not due to higher expression of CD147.

### Cu^2+^ directly interacts with the extracellular membrane-proximal domain of CD147

**Figure 4:**
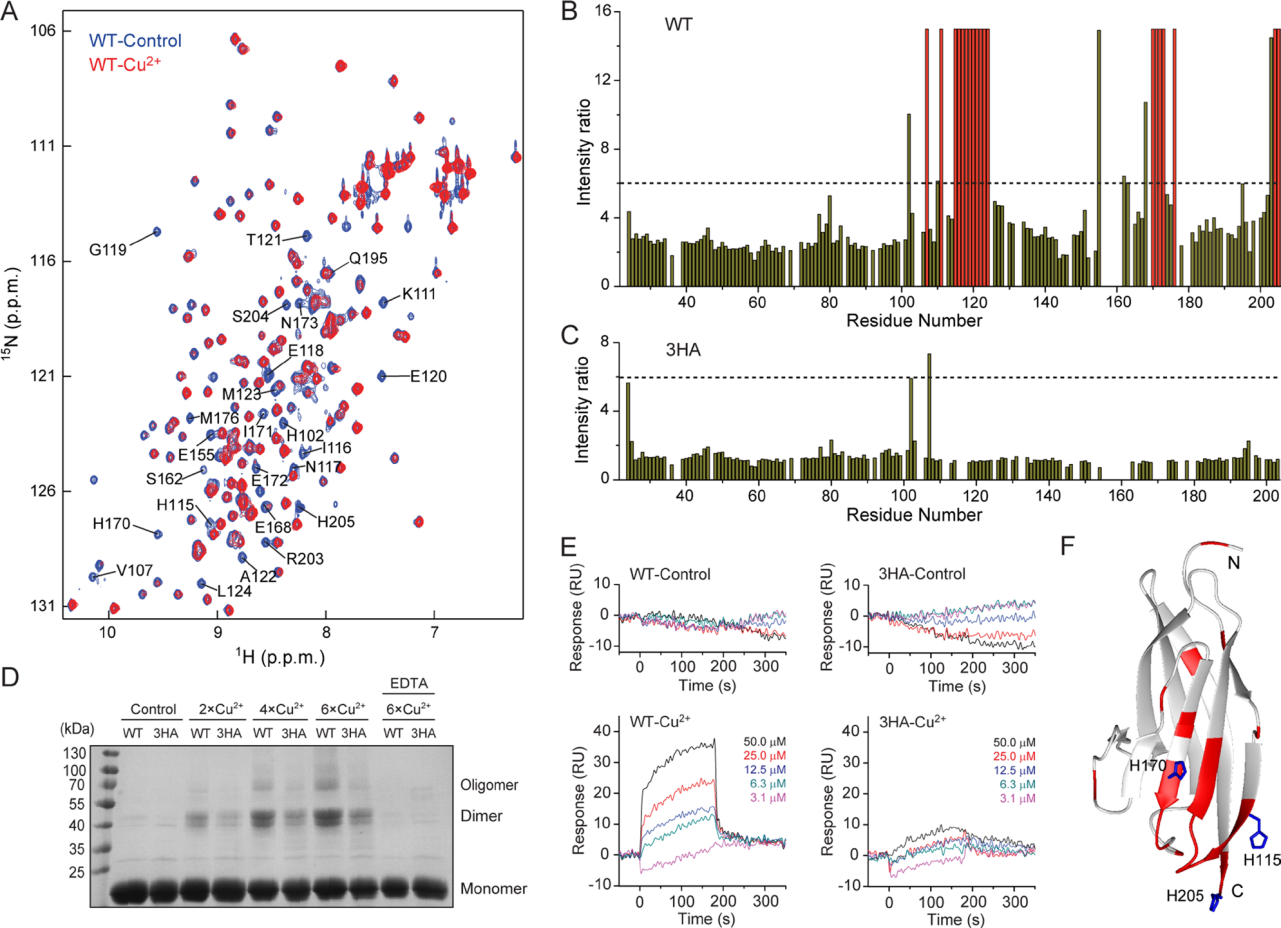
Cu^2+^ directly interacts with and mediates self-association of the membrane-proximal domain of CD147 (**A**) Overlay of 2D ^1^H-^15^N HSQC spectra of WT-CD147^ECT^ with (red) or without (blue) 2-fold excess of Cu^2+^. Residues displaying significant NH signal intensity reduction upon Cu^2+^ titration are labeled. (**B**, **C**) Plot of NH signal intensity ratio for wild-type CD147^ECT^ (B) or the 3HA mutant (C) in the absence and in the presence of 2-fold excess of Cu^2+^. Dashed lines indicate the ratio of 6. Residues with NH signals disappeared upon Cu^2+^ titration are depicted in red. (**D**) Cross-linking analysis of WT-CD147^ECT^ and the 3HA mutant with different concentrations of Cu^2+^. (**E**) SPR analysis of the homophilic interactions of WT-CD147^ECT^ or the 3HA mutant with or without Cu^2+^. RU, response unit. (**F**) Residues with missing NH signals or the signal intensity reduction ratio larger than 6 upon Cu^2+^ titration are mapped on the structure of the membrane-proximal domain of CD147. Side chains of His115, His170 and His205 are shown by sticks.

We then investigated whether the effect of Cu^2+^ is due to a direct interaction with CD147, and carried out *in vitro* NMR titration experiment with the ectodomain of CD147 (CD147^ECT^, residues 22–205). Upon titrating ^15^N-labeled CD147^ECT^ with Cu^2+^, NH signal intensities of almost all residues are gradually weakened, which could be mostly reversed with the addition of TM or EDTA (Figure 4A and 4B, Supplementary Figure 4A–4C). In the presence of 2-fold excess of Cu^2+^, there is ~2.6-fold NH signal intensity decrease relatively uniformly for residues from the membrane-distal domain (residues 22–101), and it is an average of 4.1-fold reduction for residues from the membrane-proximal domain (residues 106–205) with some NH signals showing much greater intensity decreases. NH signals of residues Val107, Lys111, His115-Leu124, His170-Asn173, Met176, Ser204 and His205 were missing, while residues His102, Glu155, Ser162, Glu168, Gln195 and Arg203 had over 6-fold NH signal intensity reduction (Figure 4A and 4B). Almost all these residues are spatially clustered on one side of the membrane-proximal domain of CD147^ECT^ (Figure 4F). The drop of NH signal intensity was presumably due to the paramagnetic relaxation enhancement (PRE) effect of Cu^2+^, as Cu^2+^ can cause enhanced relaxation of spins in proximity. This suggests a direct interaction between Cu^2+^ and CD147^ECT^.

Based on the NMR titration result, targeted mutagenesis revealed that combinations of H115A, H170A and H205A mutations progressively decreased the NH signal responses of CD147^ECT^ to Cu^2+^, while the CD147 ^ECT^ variant with triple mutations (3HA) almost abolishes the response of NMR signals to Cu^2+^ (Figure 4B and 4C, Supplementary Figure 4A and 4D). These results suggest that all three histidine residues should be involved in Cu^2+^ binding, and there may be multiple Cu^2+^ binding sites on the membrane-proximal domain of CD147 as these three residues are not near each other on protein tertiary structure (Figure 4F).

### Cu^2+^ can mediate self-association of CD147

The triple mutations should not influence the PRE effect of Cu^2+^ on NH signals from the membrane-distal domain of CD147, since all the three histidine residues are located on the membrane-proximal domain. However, the overall intensity drop caused by Cu^2+^ for NH signals of the membrane-distal domain was also significantly rescued by the 3HA mutations (Figure 4B and 4C), suggesting that the signal reduction caused by Cu^2+^ cannot be simply explained by the PRE effect. These observations prompted the speculation that Cu^2+^ can induce self-association of CD147, which results in slower rotational motion of CD147 in solution and thus faster relaxation of NMR signal.

Chemical cross-linking showed that either WT CD147^ECT^ or 3HA mutant cannot be cross-linked without Cu^2+^, consistent with that CD147 by itself is essentially a monomer in solution [26, 45]. However, in the presence of Cu^2+^, strong protein bands corresponding to CD147^ECT^ dimer and even higher order oligomers could be detected, and the amounts of cross-linked dimer and oligomers were positively correlated with the Cu^2+^ concentration (Figure 4D). The amounts of cross-linked dimer or oligomers were much less for 3HA mutant in the presence of Cu^2+^ compared to those of WT CD147^ECT^. Surface plasma resonance (SPR) further confirmed the Cu^2+^-induced self-association of CD147. No homophilic interaction among WT or 3HA mutant CD147^ECT^ proteins was observed without Cu^2+^. However, sensorgrams with clear association and dissociation processes were obtained when Cu^2+^ was present in the analyte (Figure 4E). As for 3HA mutant, the binding was dramatically reduced compared to that of WT CD147^ECT^, in the presence of Cu^2+^. These results indicate that Cu^2+^ does induce self-association of WT CD147, which is significantly impaired by the 3HA mutations.

### Cu^2+^-mediated CD147 self-association up-regulates MMPs expression

**Figure 5:**
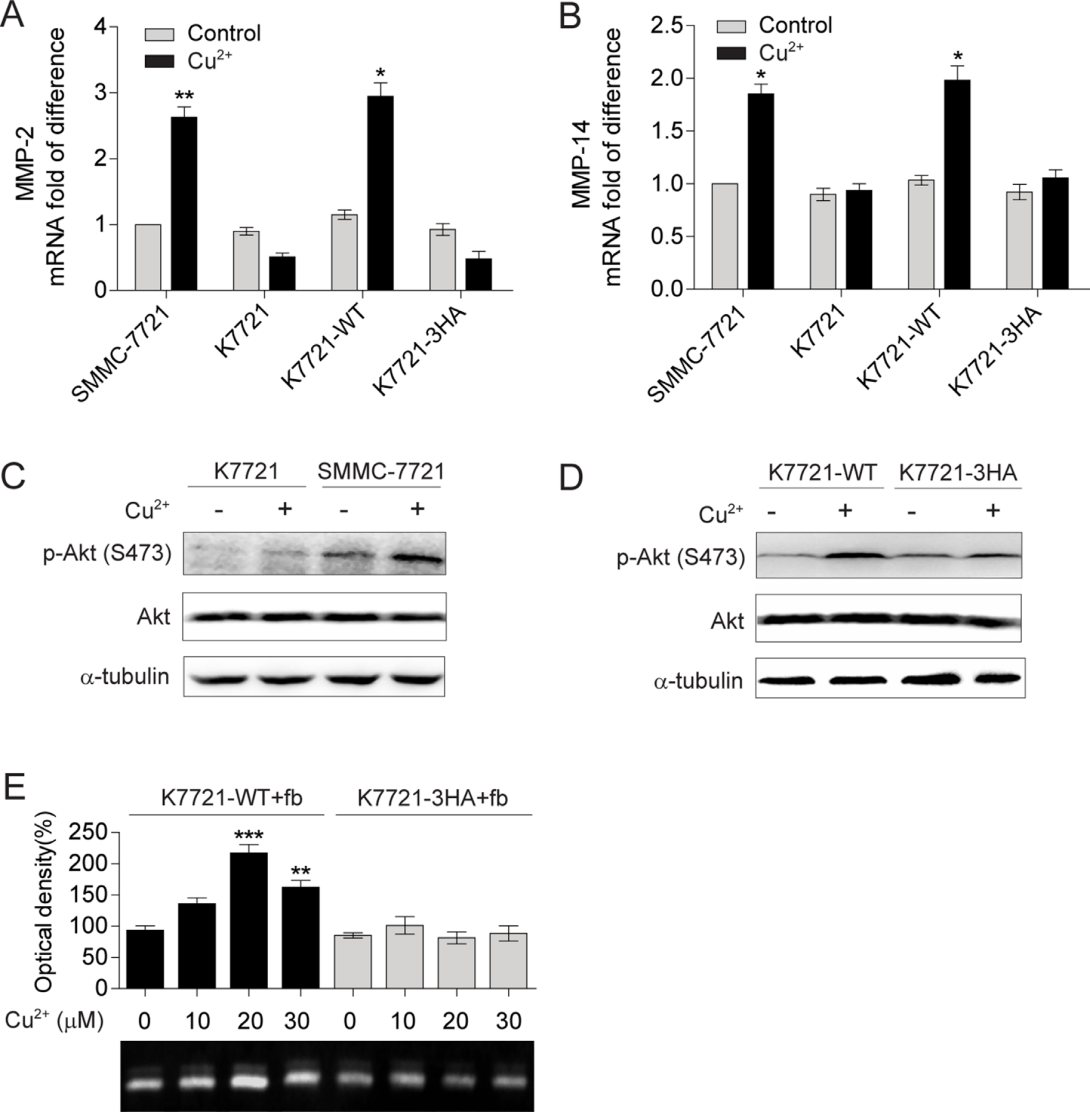
Self-association of CD147 mediated by Cu^2+^ up-regulates MMP-2 and MMP-14 expression (**A**, **B**) qRT-PCR analysis of MMP-2 (A) and MMP-14 (B) in SMMC-7721, K7721, K7721-WT and K7721-3HA cells with or without Cu^2+^ treatment (30 μM for MMP-2 detection and 20 μM for MMP-14 detection, *n* = 3, mean ± SEM, *t*-test). (**C**, **D**) Immublot analysis of the p-Akt levels in SMMC-7721, K7721 cells (C) and in K7721-WT, K7721-3HA cells (D) with or without 10 μM Cu^2+^ treatment. (**E**) Gelatin zymogrphy analysis of MMP-2 secretion from fibroblasts (fb) co-cultured with K7721-WT and K7721-3HA cells treated with different concentrations of Cu^2+^ (*n* = 3, mean ± SEM, one way ANOVA). Gel images in panel C and D are representative of at least two technical replicates.

To investigate the role of Cu^2+^-induced CD147 self-association, WT or 3HA mutant CD147 was stably transfected into *cd147*^—/—^ SMMC-7721 (K7721) cells. The resulting cell lines with WT CD147 (K7721-WT) or 3HA mutant (K7721-3HA) expressed on cell surfaces had similar expression levels to that of SMMC-7721 cells (Supplementary Figure 5). It is interesting to notice that all four types of cells (SMMC-7721, K7721, K7721-WT and K7721-3HA) showed similar low expression levels for MMP-2 and MMP-14 (Figure 5A and 5B), without the addition of Cu^2+^, suggesting that Cu^2+^ is required for CD147 to induce MMPs production.

Distinct from those of SMMC-7721 cells, MMP-14 mRNA level was not changed and that of MMP-2 was even reduced in K7721 cells when treated with Cu^2+^, which can be fully rescued by the expression of WT CD147 but not the 3HA mutant (Figure 5A and 5B). Consistently, Cu^2+^ was unable to activate the PI3K/Akt signaling in K7721 cells (Figure 5C), and the expression of WT CD147 rather than the 3HA mutant can restore the Cu^2+^ augmented p-Akt level (Figure 5D). Gelatin zymography also showed that MMP-2 production was not significantly changed in Cu^2+^ treated fibroblasts co-cultured with K7721-3HA cells, while that for K7721-WT cells was increased by 2.2-fold with 30 μM Cu^2+^ treatment (Figure 5E). As the 3HA mutations nearly abolish the binding of Cu^2+^ to CD147^ECT^ and impair the Cu^2+^-induced oligomerization of CD147^ECT^, these results suggest that mediating CD147 self-association is the underlying molecular mechanism for Cu^2+^ to activate the PI3K/Akt signaling and stimulate MMP-2 and MMP-14 expression.

To further test whether the intracellular domain of CD147 is involved in the signal transduction for extracellular Cu^2+^, a K7721 cell line that expressed a truncated version CD147 (K7721-ΔC) with its intracellular domain (residues 229–269) deleted was constructed. It was found that extracellular Cu^2+^ was still able to up-regulate MMP-2 and MMP-14 expression in K7721-ΔC cells (Supplementary Figure 6), suggesting that the intracellular domain of CD147 is not required for the signal transduction of this process. It is possible that the signal of Cu^2+^-mediated CD147 oligomerization is transduced into the cell through interactions with other cell surface proteins, yet to be identified.

### Cu^2+^-mediated CD147 self-association accelerates HCC cell invasion

**Figure 6:**
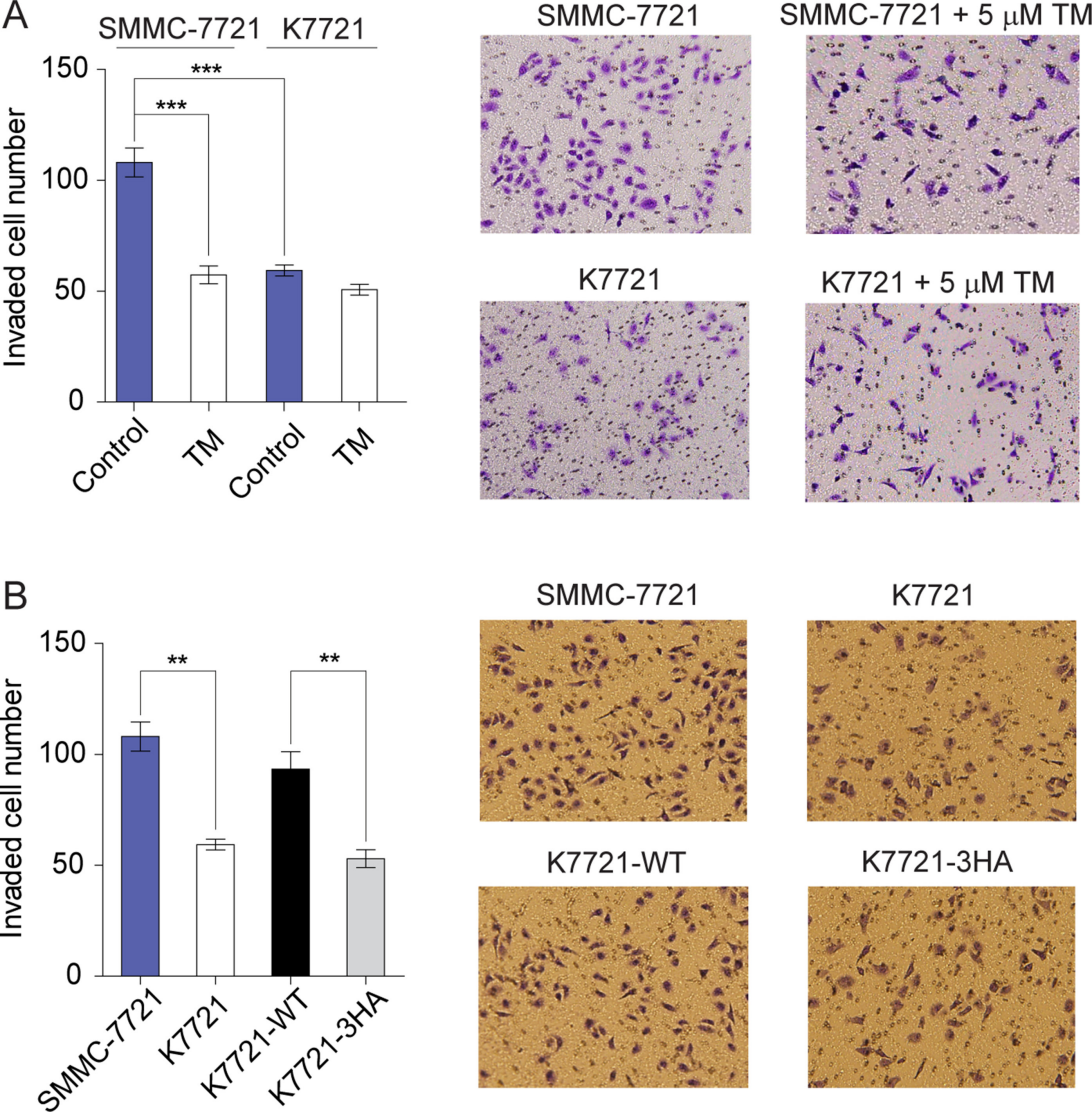
Cu^2+^-mediated CD147 self-association enhances HCC cells invasiveness (**A**) *In vitro* invasion assay of SMMC-7721 and K7721 cells with or without 5 μM TM treatment (*n* = 3, mean ± SEM, one way ANOVA). (**B**) Invasion assay of SMMC-7721, K7721, K7721-WT and K7721-3HA cells (*n* = 3, mean ± SEM, one way ANOVA). **P < 0.01, ***P < 0.001.

CD147 and MMPs, especially MMP-2 and MMP-14, are frequently involved in tumor cell metastasis [30, 35]. Meanwhile, the copper chelator TM was reported to significantly decrease tumor cell invasiveness [39]. To assess whether self-association of CD147 mediated by Cu^2+^ can modulate the invasion ability of tumor cells, transwell invasion assays were performed. It was found that the invasiveness of K7721 cells was dramatically decreased compared with SMMC-7721 cells (Figure 6A). The copper chelator TM inhibited the invasion ability of SMMC-7721 cells, while it had no effect on K7721 cells (Figure 6A). Moreover, the expression of WT CD147 but not the copper-binding deficient 3HA mutant can restore the reduced invasiveness of K7721 cells due to CD147 knockout (Figure 6B). These results indicate that self-association of CD147 enhances the invasiveness of SMMC-7721 cells, which can be abolished either by the copper chelator TM or by the 3HA mutations. Thus, Cu^2+^-mediated CD147 self-association may play an important role in HCC cell metastasis.

## DISCUSSION

Recent studies revealed that CD147 also functions as the receptor for multiple protein factors, such as chemoattractant CyPA [24, 46, 47], malaria invasion protein RH5 [25, 26], meningococcal pilus components PilE and PilV [27], and Rod-derived cone viability factor (RdCVF) [28]. Here, we have demonstrated that CD147 is a bona fide signaling receptor for extracellular Cu^2+^ in cancer cells: the self-association of CD147 mediated by extracellular Cu^2+^ signals the up-regulation of MMP-2 and MMP-14 in HCC cells through activating PI3K/Akt pathway, stimulates MMP-2 production from neighboring fibroblasts and promotes HCC cell invasion. Without CD147, extracellular Cu^2+^ did not show a stimulating role on MMP-2 and MMP-14 expression, as well as PI3K/Akt signaling. Consistently, Cu^2+^ only has a marginal effect on MMPs induction when CD147 expression level is knockdown with shRNA. Meanwhile, without extracellular Cu^2+^, whether the expression of CD147 or not does not affect the expression of MMP-2 and MMP-14, neither the invasiveness of HCC cells. It may provide an explanation for a recent report that claims CD147 is unable to induce the production of MMPs [48]. Therefore, the MMPs-inducing activities of CD147 and Cu^2+^ are interdependent, and the roles of copper and CD147 in cancer progression are unprecedentedly linked together.

As an essential trace metal for life, copper should be involved in different aspects of cancer, and emerging evidences suggest that copper is a key regulator of many cell signaling pathways [49]. While most previous studies were focused on the intracellular roles of copper [1, 18–20], the biological roles of extracellular Cu^2+^ in cancer have been largely ignored. Although it is well established that most of serum copper is incorporated into ceruloplasmin (CP), the finding of intracellular copper translocation across cell membrane from recent studies make it possible that transient high concentration of free copper outside the plasma membrane may exist physiologically [3, 22, 23]. Our results clearly demonstrate that extracellular Cu^2+^ plays a direct signaling role through its cell surface receptor CD147 in HCC cells. We found that copper can also up-regulate MMP-2 and MMP-14 expression in human hepatocellular carcinoma cell HUH-7 and human pancreatic cancer cell MIA PaCa-2 (Supplementary Figure 7). It is thus very likely that the signaling role of extracellular Cu^2+^ may also apply to other type of cancer cells with high CD147 expression level. As copper, CD147, MMP-2 and MMP-14 are all implicated to promote the metastasis of cancer [3, 32, 35], our study sheds light upon their functional mechanisms and relationship in cancer progression. Therefore, targeting the interaction between Cu^2+^ and CD147 or the Cu^2+^-induced self-association of CD147 may provide an alternative therapeutic strategy to anti-cancer drug development. Meanwhile, as we have found that TM has no effect on the invasiveness of cancer cells without CD147, it may provide a strategy of precision medicine for patient selection in the design of future clinic trials for copper-lowering anti-cancer therapy.

## MATERIALS AND METHODS

### Cell culture

Human hepatocellular carcinoma cells SMMC-7721 and human fibroblast cells were obtained from the Institute of Cell Biology, Academic Sinica (Shanghai, China). Human hepatocellular carcinoma cells HuH-7 were purchased from the Japanese Collection of Research Bioresources Cell Bank (JCRB). Human pancreatic cancer cells MIA PaCa-2 were purchased from the American Type Culture Collection (ATCC). K7721 (*cd147*^—/—^ SMMC-7721), SMMC-7721 shCD147, SMMC-7721 snc, MIA PaCa-2 shCD147 and MIA PaCa-2 snc cells were developed and preserved in our laboratory.

SMMC-7721, SMMC-7721 shCD147, SMMC-7721 snc, K7721 cells and fibroblasts were cultured in RPMI 1640 medium (Gibco, New York, USA). HuH-7, MIA PaCa-2, MIA PaCa-2 shCD147 and MIA PaCa-2 snc cells were cultured in DMEM medium (Gibco, New York, USA). Both media were supplemented with 10% FBS and 1% penicillin/streptomycin and all cells were cultured at 37°C with 5% CO_2_.

### RNA interference

siRNAs were transfected into SMMC-7721 cells using Lipofectamine 2000 reagent according to the manufacturer's instructions (Invitrogen, Carlsbad, CA). siRNAs for human CTR1 and silencer negative control siRNA (sncRNA) were purchased from Santa Cruz (Dallas, Texas).

### Plasmids transfection

pcDNA3.1 encoding full-length WT-CD147 or 3HA mutant was transfected into K7721 cells using Lipofectamine 2000 reagent according to the manufacturer's instructions (Invitrogen, Carlsbad, CA). After G418 selection, the cells were sorted by flow cell sorter.

### qRT-PCR

SMMC-7721 or other Cells (5×10^5^) were seeded and incubated in 6-well plates until the cell density was 80%. Cells were rinsed three times with serum-free medium and incubated in serum-free medium for 24 h. Then, cells were washed three times again and serum-free medium with different concentrations of Cu^2+^ was added for further incubation of 24 h. Total RNA was extracted using a total RNA extraction Kit (Omega, Norcross, GA) and reversely transcribed with a ReverTra Ace-a kit (Toyobo, Osaka, Japan). Real-time RT-PCR was performed using the SYBR Premix EX Taq II (Takara, Shiga, Japan) with the sequence detection system Stratagene Mx3005P (Agilent Technologies, Frankfurt am Main, Germany). The primers were synthesized by BGI (Beijing, China) and are listed in Supplementary Table 1. GAPDH was used to normalize the RNA inputs, which yield ΔCt values. All the mRNA fold of difference values (2^-ΔΔCt) were relative to the control group (SMMC-7721 cells without Cu2+ treatment), while ΔΔCt was calculated by subtracting ΔCt value of the control group. Errors were assessed for fold of difference values from 3 independent experiments.

### Proliferation assay

SMMC-7721 cells (5 × 10^4^) were seeded in 96-well plates and incubated overnight. Cells were rinsed three times with serum-free medium and incubated in serum-free medium for 24 h. Then, cells were washed three times again and serum-free medium with different concentrations of Cu^2+^ was added for further incubation of 24 h. 10 μL/well WST-1 was added and incubated for 2 h. The absorbance of the dye with a wavelength of 450 nm was then detected by a scanning multiwell spectrophotometer (Bio-Rad, Hercules, CA). Culture medium with WST-1 was used as blank control. Three different experiments were performed for each experimental condition.

### Atomic absorption spectroscopy

SMMC-7721 cells transfected with siRNA for CTR1 or sncRNA were treated with different concentrations of Cu^2+^ for 24 h and then trypsinized, rinsed three times with PBS. Cells were harvested and digested with 30 μL 65% nitric acid at 60°C for 2 h. Samples were diluted 3 000 times with deionized water and the copper concentrations were determined with flame atomic absorption spectrometer (ZEEnit700P, Analytik Jena).

### Western blotting

Cells were lysed with RIPA cell lysis buffer (Beyotime, Shanghai, China), and the protein quantification was determined by BCA Protein Assay Kit (Pierce Biotechnology, Rockford, IL). Equal quantities of protein were resolved by 10% SDS-PAGE and transferred to polyvinylidene fluoride (PVDF) microporous membrane (Millipore, Boston, MA). After being blocked with 5% fat-free milk, the membrane was probed with primary antibodies including rabbit anti-MMP-14 (Abcam, ab51074), HAb18 against CD147 (prepared by our laboratory), rabbit anti-CTR1 (Epitomics, 5773-1), rabbit anti-AKT (Cell Signaling Technology, 4685), rabbit anti-phospho(S473)-Akt (Abcam, ab81283). Following incubation with horseradish peroxidase-conjugated goat anti-rabbit or anti-mouse IgG (Santa Cruz, sc-2004 and sc-2005), protein bands on the membrane were visualized using a chemiluminescence kit (Beyotime, Shanghai, China) according to the manufacturer's instructions. Mouse antibody against α-tubulin (Abcam, ab80779) or β-actin (Abcam, ab6276) was used to control for differences in protein loading.

### Gelatin zymography

SMMC-7721 (or MIA PaCa-2) cells and fibroblasts were co-cultured in serum-free medium with different concentrations of Cu^2+^. The conditioned medium was collected and separated by 10% SDS-PAGE gels containing 0.1% gelatin for 30 min twice. The gels were washed in a 2.5% Triton X-100 (Sigma) solution at room temperature with gentle agitation. The gels were then soaked in reaction buffer containing 50 mM Tris-HCl (pH 7.3), 200 mM NaCl, and 5 mM CaCl_2_ at 37°C for 24 h. The gels were stained with Coomassie Brilliant Blue R250 for 2 h and destained for 0.5 h. The zones of gelatinolytic activity were revealed by negative staining.

### Protein purification

DNA sequence encoding the extracellular portion of CD147 (residues 22–205) was subcloned into the pET21a vector (Novagen). Point mutations were generated using the site-directed mutagenesis kit (SBS Genetech). *Escherichia coli* strain Origami (DE3) (Novagen) harboring the construct was cultured in LB medium at 35°C until OD_600_ > 1.0, and protein expression was induced with 100 μM IPTG at 18°C. For ^15^N isotopically labeling, the bacteria were first grown in LB medium till OD_600_ > 1.0, then collected and resuspended in ^15^N-labeled M9 minimal medium for continuing growth, and 100 μM IPTG was added to induce protein expression after 40 min at 18°C. The Cells were harvested by centrifugation 24 h after induction and resuspended in lysis buffer (50 mM Tris-HCl, 40 mM NaCl, pH 8.5), then lysed by freezing and thawing, followed by sonication. After centrifugation, the supernatant was applied to ion-exchange column with DEAE-cellulose DE52 (Whatman). Protein was further purified with size exclusion chromatography on a superdex 75 column (Amersham) in 50 mM Tris-HCl with 50 mM NaCl (pH 7.5).

### NMR titration experiment

The NMR samples all contained 0.4 mM uniformly ^15^N labeled protein in 50 mM Tris-HCl, 50 mM NaCl (pH 7.5) with 90% H_2_O/10% D_2_O. A series of 2D ^1^H-^15^N HSQC spectra with gradually increased Cu^2+^ concentration (0.2 mM, 0.4 mM, 0.8 mM) were collected at 298 K on a Bruker Avance 500 MHz spectrometer with a triple-resonance cryoprobe. An excess of TM or EDTA (4 mM) was added to the NMR sample for the final 2D ^1^H-^15^N HSQC spectrum.

### Cross-linking

Protein (75 μM) with or without Cu^2+^ (150 μM, 300 μM or 450 μM) was cross-linked with 150 μM ethylene glycolbis (succinimidyl succinate) (EGS) (Pierce) in the reaction buffer (20 mM MOPS, 50 mM NaCl, pH 7.2). Before cross-linking by EGS, EDTA was added with the final concentration of 8 mM when it was used. The reaction mixture was incubated at room temperature for 30 min, and then the reaction was quenched by adding Tris (1 M, pH 7.5) to a final concentration of 100 mM.

### Surface plasma resonance

Surface plasmon resonance experiments were performed using the ProteOn^TM^ XPR36 protein interaction array system (Bio-Rad). Briefly, bait CD147 was immobilized on a ProteOn^TM^ GLC sensor chip (Bio-Rad) by amine coupling. A concentration series of CD147 as analyte (3.125, 6.25, 12.5, 25 and 50 μM) with or without 400 μM Cu^2+^ was injected over the CD147-coated chip for 180 s at 50 μL/min, followed by a 720 s dissociation time. The chip surface was then regenerated with a pulse of 2 M NaCl at the end of each cycle. All experiments were performed at 25°C in 20 mM Tris-HCl (pH 7.5), 200 mM NaCl, 0.005% Tween 20.

### Transwell invasion assay

Chambers with polycarbonate filters with 8-mm nominal pore size (Millipore) coated on the upper side with Matrigel (BD Bioscience, San Jose, CA) was used to assess cell invasiveness. The chambers were placed into a 24-well plate. Cells were trypsinized, resuspended in serum-free media, and seeded at 1×10^5^ cells per well in the upper chamber. The lower chamber was filled with 500 μL 1640 medium containing 15% FBS with different concentrations of copper chelator TM. Following 16 h incubation, cells remaining in the upper chamber were completely removed by gently swabbing. The cells that passed through the filter and invaded the lower chamber were stained by crystal violet and counted.

## SUPPLEMENTARY MATERIALS FIGURES AND TABLES



## Abbreviations

HCC: hepatocellular carcinoma
TM: tetrathiomolybdate
MMP: matrix metalloproteinases
PI3K: phosphoinositide 3 kinase
qRT-PCR: quantitative PCR with reverse transcription
NMR: nuclear magnetic resonance
PRE: paramagnetic relaxation enhancement
SPR: surface plasma resonance
