# Supplementary material for: CD147 functions as the signaling receptor for extracellular divalent copper in hepatocellular carcinoma cells
Source: Oncotarget. 2017 May 9;8(31):51151–63. doi: 10.18632/oncotarget.17712 (PMC5584238; doi:10.18632/oncotarget.17712)
Supplement: Supplementary file 1 [file oncotarget-08-51151-s001.pdf]

CD147 functions as the signaling receptor for extracellular divalent copper in hepatocellular carcinoma cells

SUPPLEMENTARY MATERIALS

Supplementary Table 1: Primers used for RT-PCR

Genes	Forward	Reverse
mmp-1	GAAGAATGATGGGAGGCAAGT	GAGGACAAACTGAGCCACATC
Mmp-2	GGCAGTGCAATACCTGAACAC	GTCTGGGGCAGTCCAAAGAACT
Mmp-3	TTTTACCCTTTTGATGGACCTG	GTCCCTGTTGTATCCTTTGTCC
Mmp-9	TTCCCCTTCACTTTCCTGGGTA	CGCCACGAGGAACAAACTGTAT
Mmp-14	CTTTTCCATCCCCTGACATACC	CTGACTGAGCAACGAAGACCCT
Gapdh	TGATGACATCAAGAAGGTGGTGAAG	TCCTTGGAGGCCATGTGGGCCAT

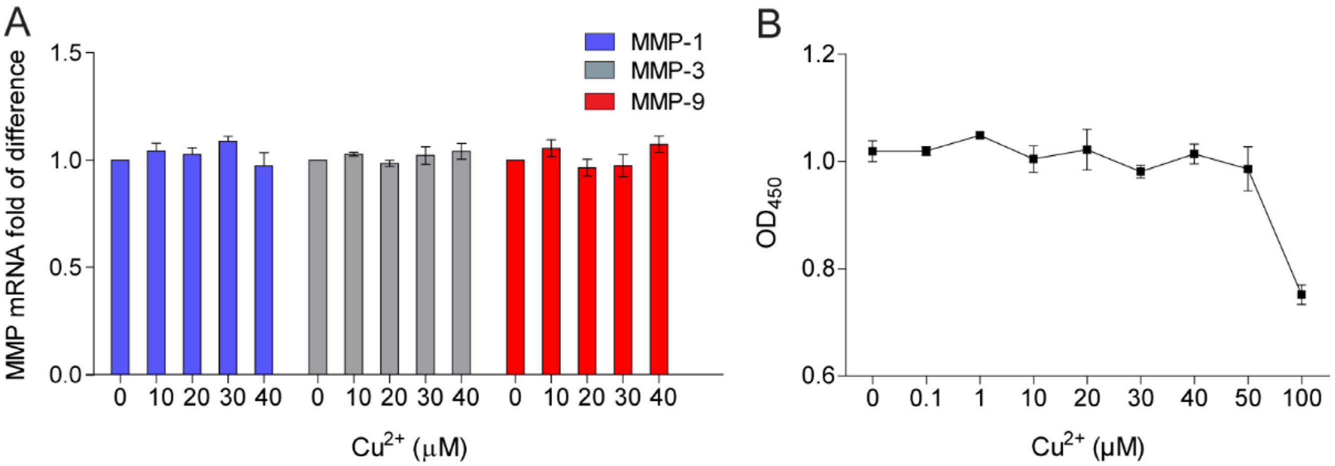

Supplementary Figure 1: Copper has no effect on the expression of MMP-1, MMP-3 and MMP-9 in SMMC-7721 cells. (**A**) qRT-PCR analysis of MMP-1, MMP-3 and MMP-9 in SMMC-7721 cells treated with different concentrations of Cu^{2+}. (**B**) Effect of copper on the viability of SMMC-7721 cells as assessed by WST-1 assay. Up to 50 μM, copper does not significantly affect the cell viability. All data are presented as mean ± SEM. of three independent experiments.

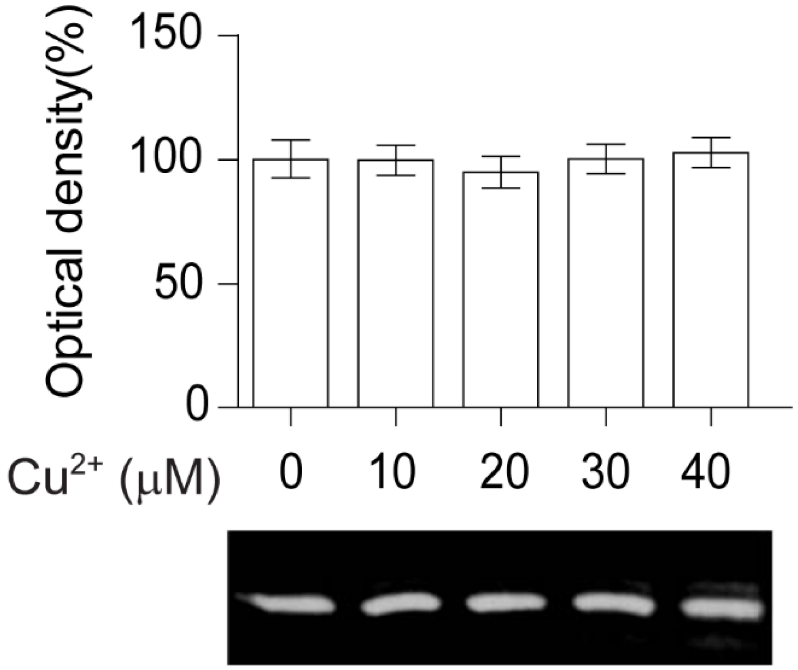

Supplementary Figure 2: MMP-2 expression of fibroblasts treated with Cu^{2+}. Data are presented as mean ± SEM (n = 3). One representative gelatin zymography is shown.

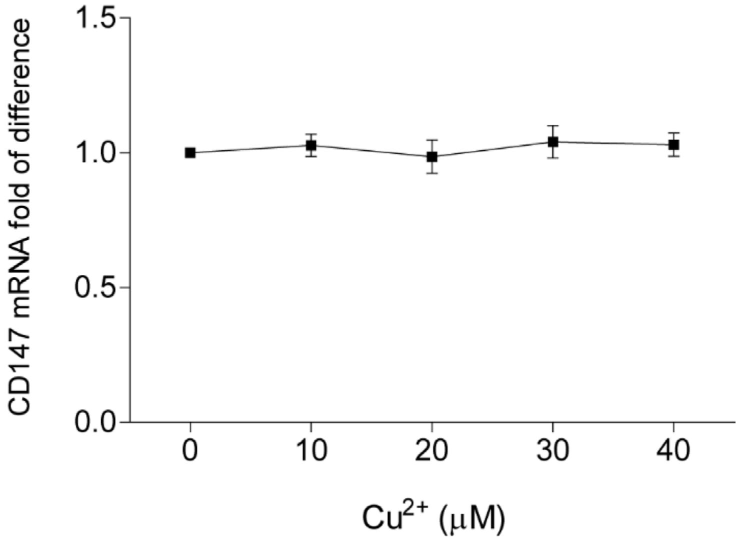

Supplementary Figure 3: Copper does not affect the expression of CD147. qRT-PCR analysis of CD147 expression in SMMC-7721 cells treated with different concentrations of Cu^{2+}. Data are presented as mean ± SEM of three independent experiments.

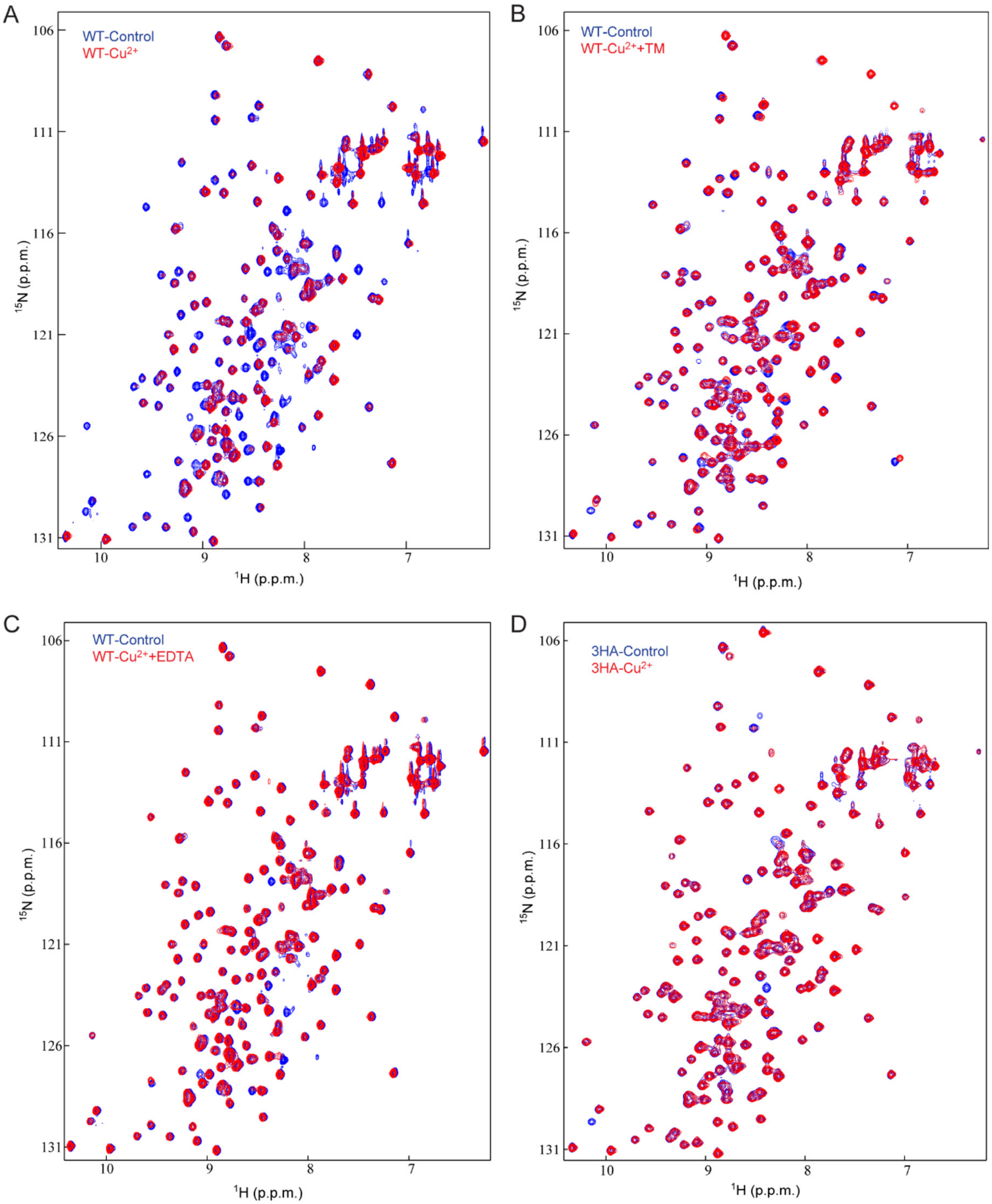

Supplementary Figure 4: NMR titration experiments of WT CD147 or the 3HA mutant. (**A**) Overlay of 2D ^{1}H-^{15}N HSQC spectra of WT-CD147^{ECT} with (red) or without (blue) 2-fold excess of Cu^{2+}. (**B**, **C**) NH signal intensity reduction caused by Cu^{2+} can be rescued by copper chelator TM (B) or EDTA (C). (**D**) Overlay of 2D ^{1}H-^{15}N HSQC spectra of the 3HA mutant with (red) or without (blue) 2-fold excess of Cu^{2+}.

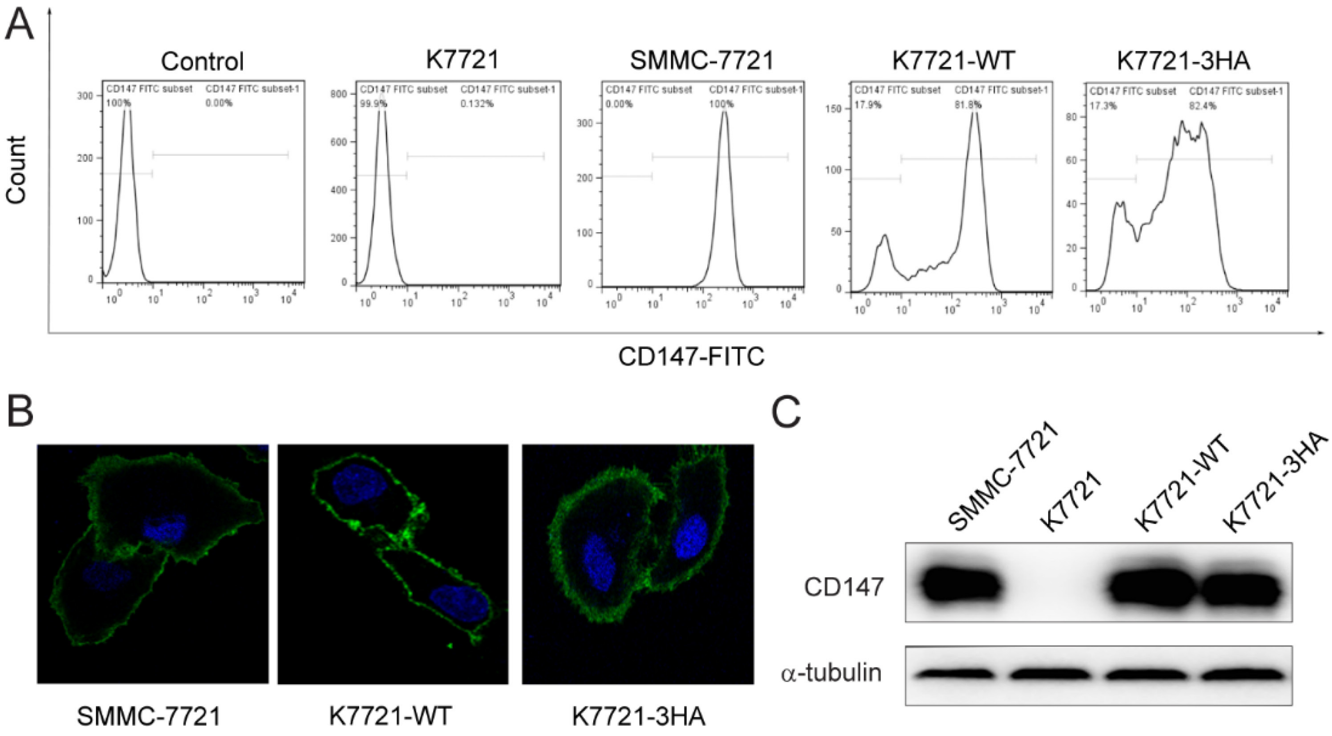

Supplementary Figure 5: Expression of CD147 in SMMC-7721, K7721-WT and K7721-3HA cells. (**A**)CD147 expressed in SMMC-7721, K7721-WT and K7721-3HA cells, but not K7721 cells. (**B**) CD147 expressed uniformly on the plasma membrane of SMMC-7721, K7721-WT and K7721-3HA cells as imaged by immunofluorescence. (**C**) Immunoblot analysis of CD147 in SMMC-7721, K7721, K7721-WT and K7721-3HA cells.

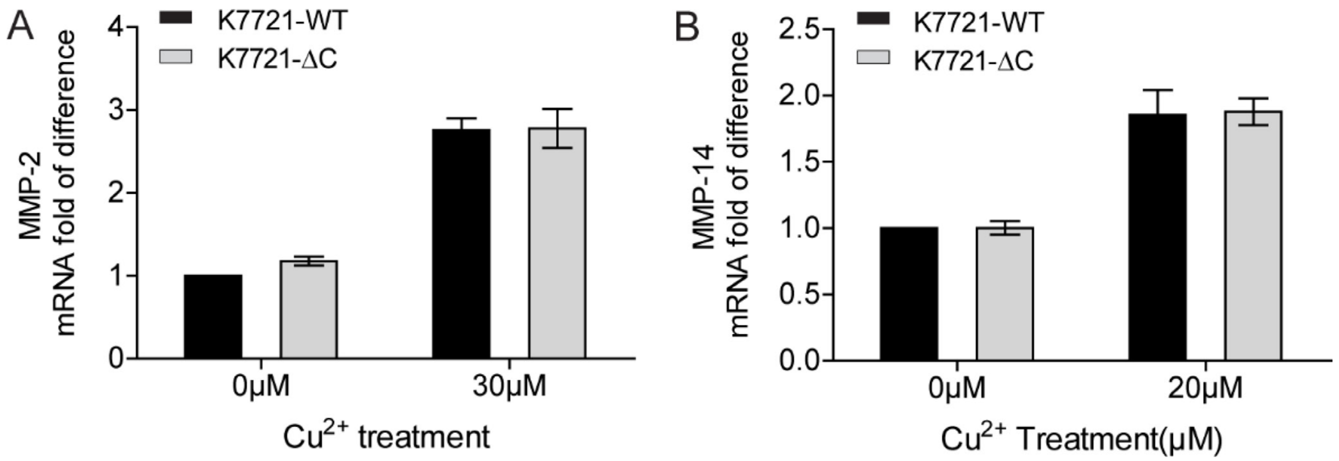

Supplementary Figure 6: The intracellular domain of CD147 is not required for MMP up-regulation by extracellular Cu^{2+}. (**A**, **B**) qRT-PCR analysis of MMP-2 (A) and MMP-14 (B) in K7721-WT and K7721-ΔC cells with or without Cu^{2+} treatment (n = 3, mean ± SEM).

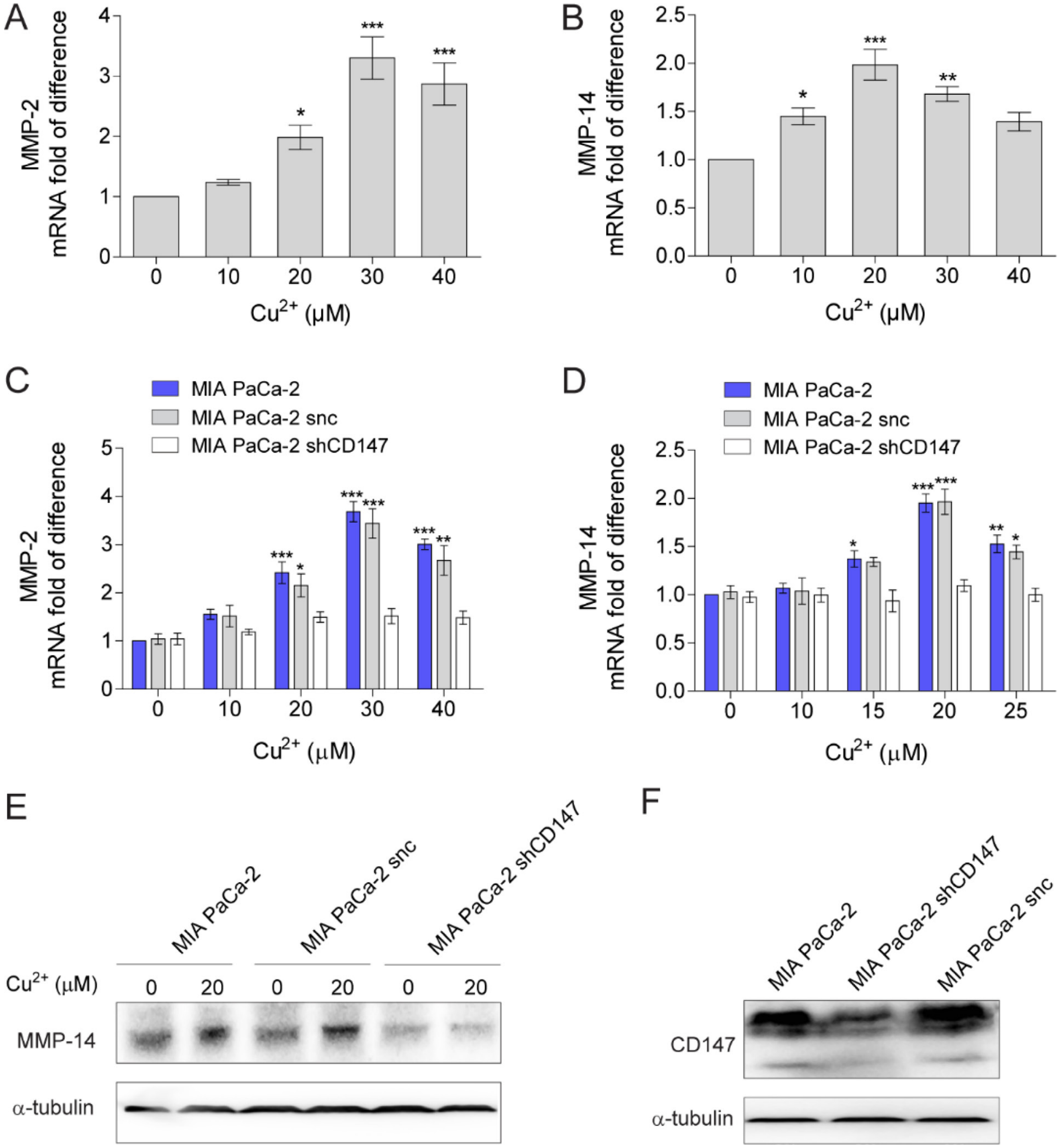

Supplementary Figure 7: Copper up-regulates the expression of MMP-2 and MMP-14 in HUH-7 and MIA PaCa-2 cells. (**A**, **B**) qRT-PCR analysis of MMP-2 (A) and MMP-14 (B) in HUH-7 cells treated with different concentrations of Cu^{2+} ($n = 3$, mean ± SEM, one way ANOVA). (**C**, **D**) qRT-PCR analysis of MMP-2 (C) and MMP-14 (D) in MIA PaCa-2 cells treated with different concentrations of Cu^{2+} (n=3, mean ± SEM, one way ANOVA). Cells transfected with *cd147*-specific shRNA (MIA PaCa-2 shCD147) or control shRNA (MIA PaCa-2 snc) were also analyzed. (**E**). Immunoblot analysis of MMP-14 in MIA PaCa-2, MIA PaCa-2-NC or MIA PaCa-2-shCD147 cells with or without Cu^{2+} treatment. (**F**) The expression of CD147 is knocked down in MIA PaCa-2 shCD147 cells. *$P < 0.05$, **$P < 0.01$ and ***$P < 0.001$. Gel images in panel E and F are representative of at least two technical replicates.
